# Evaluation of the insecticide susceptibility profile in *Cimex lectularius* (Hemiptera: Cimicidae) in Belo Horizonte (Brazil)

**DOI:** 10.1590/0037-8682-0707-2020

**Published:** 2021-11-15

**Authors:** Grasielle Caldas D’Ávila Pessoa, Diego Oliveira da Silva, Aline Cristine Luiz Rosa, Pedro Horta Andrade, Liléia Diotaiuti

**Affiliations:** 1 Universidade Federal de Minas Gerais, Instituto de Ciências Biológicas, Departamento de Parasitologia, Laboratório de Fisiologia de Insetos Hematófagos, Belo Horizonte, MG, Brasil.; 2 Fundação Oswaldo Cruz, Instituto René Rachou, Grupo de Pesquisa Triatomíneos, Belo Horizonte, MG, Brasil.

**Keywords:** Heteroptera, Control, Insecticide resistance

## Abstract

**INTRODUCTION:**

Since 2013, major *Cimex lectularius* infestations have been detected in public shelters in Belo Horizonte (Brazil). Due to this, insecticide resistance has been investigated as one of the possible causes for the failure to control bedbugs.

**METHODS:**

Cimicids were subjected to bioassays according to the World Health Organization recommendations using deltamethrin and all commercial insecticides available for control of *Cimex* in Brazil.

**RESULTS:**

Cimicids were deltamethrin resistant and presented indicative of resistance to other insecticides, except for propoxur 1%.

**CONCLUSIONS:**

The commercial insecticides have a limited effect on bedbug populations, which may justify the unsatisfactory control observed in the shelters studied.

The Cimicidae family comprises six subfamilies, 24 genera, and 110 species, the vast majority of which are ectoparasites that feed exclusively on bats and birds. Only *Cimex lectularius* and *Cimex hemipterus* are known to be anthropophilic. Although species are cosmopolitan, *C. lectularius* and *C. hemipterus* are most commonly found in tropical and temperate regions, respectively. It should be noted that *Leptocimex boueti*, primarily associated with bats, is a species that regularly feeds on humans, but is restricted to West Africa^1^ ^,^ ^2^. 

*C. lectularius and C. hemipterus* have already been found to be naturally infected with *Wuchereria bancrofti*, *Brugia malayi, Trypanosoma cruzi*, *Plasmodium* sp.*, Leishmania* sp., *Brucella melitensis*, *Coxiella burnetii* and *Rickettsia prowazecki*; they also carry the hepatitis B virus and human immunodeficiency virus. In addition, under laboratory conditions, these insects have been shown to be susceptible to infection by more than 65 species of microorganisms, including fungi, viruses, bacteria and protozoa. However, in the absence of robust scientific evidence, the natural transmission of pathogens by bed bugs is still a matter of debate^3^.

On the other hand, dermatitis caused by the saliva of cimicids exerts an undeniable impact on the physical, mental, and emotional health of humans ectoparasitized by bed bugs. In practice, bedbug bites cause intense discomfort during sleep, which can result in lethargy, anxiety, physical and psychological stress, and insomnia. Although the bites are painless, they result in dermatitis accompanied by itching and allergies of varying intensity. In the case of more severe infestations, especially when the bites affect children, impaired individuals, and/or the elderly, cimicids are suspected to cause vitamin deficiencies and anemia because of the volume of blood extracted from the hosts^3^ ^,^ ^4^.

In Belo Horizonte (Minas Gerais State/Brazil), in 2013 and 2014, a large infestation of bed bugs was detected in the homeless shelter “Albergue Tia Branca (ATB)” and at the migrant shelter “Pousadinha Mineira (PM)”. Despite structural renovations made in these environments (e.g., replacement of wooden beds with metal beds, with galvanized sheet layers; replacement of infested mattresses with new ones; sealing of cracks in beds and walls, etc.) associated with the adoption of chemical control strategies (use of pyrethroid insecticides), infestations were not controlled. In this context, this study investigated the resistance of these populations of *C. lectularius* to insecticides, including the formulated insecticides available in Brazil for the control of cimicids and/or vectors of medical importance, as one of the possible causes for the failure of activities adopted in the infested shelters to control the infestations. 

Bed bugs were collected in 2014 and 2015 at the ATB (-19.9207489, -43.9306985) and PM (-19.9199368, -43.9374821) shelters and were identified according to Forattini^1^. Cimicid colonies were established in the insectarium of the Reference Laboratory for Triatomines and Epidemiology of Chagas Disease (Instituto René Rachou/FIOCRUZ) under controlled temperature and humidity conditions (25 °C ± 1 °C; 60% ± 10% RH). Feeding was offered twice a week to anesthetized hairless mice using 10% ketamine and 2% xylazine (CEUA no. 97/2016). 

Laboratory bioassays were carried out in accordance with the World Health Organization recommendations^5^. *C. lectularius* (5-day-old, post-molt adults; 72 hours after blood feeding) were exposed for 24 h to impregnated papers with the insecticides of interest. The susceptibility to technical grade deltamethrin (Bayer, 99.1% pure) was evaluated at a concentration of 0.132 mg/cm^2^, diluted in chloroform P.A. and Vaseline. The insects in the control group were exposed to papers impregnated with only chloroform P.A. with Vaseline (C1). Additionally, the cimicids were exposed to eight commercial insecticide formulations allowed in Brazil to control bed bugs and/or other urban pest/disease vectors, looking for the possibility of being used for cimicid control (Table 1). The products were diluted in water to the concentrations recommended by the manufacturers. The insects in the control group were exposed to papers treated with water (C2). The tests were carried out with 10 insects per dose, in triplicate, on independent days. After 24 h of exposure to the insecticide-impregnated papers, the cimicids were transferred to Petri dishes covered with filter paper without insecticide. After 24 h, the mortality rate was measured. Insects were considered alive when unchanged locomotor activity was observed, without signs of paralysis and/or a knockdown effect. The populations whose mortality to the insecticide was equal to or lower than 98% or greater than 98% were considered resistant and susceptible, respectively^5^. The statistical analyses were performed using the R program (3.3.2 version) considering the data normality. 

**TABLE 1:** Commercial insecticide formulations used in laboratory bioassays to characterize the susceptibility/resistance of *Cimex lectularius* from Belo Horizonte, MG (Brazil).

*Commercial insecticide name*	*Chemical composition*	*Recommendation for use*	*Recommended dose for use*	*Manufacturer*
Bifentol® 200 SC ^1^	Bifenthrin 20%	Bed bugs	2.5 mL/L	Chemone
Plurestro® PRO Aerosol^1^	Pyrethrin 0.5%	Bed bugs	-	Basf
Calira® Aerosol ^2^	Propoxur 1%	Bed bugs	-	Basf
Tenopa® SC ^3^	Alpha-Cypermethrin 3% with Flufenoxuron 3%	Bed bugs	17 mL/L	Basf
Temprid® SC ^4^	Beta-Cyfluthrin 10.5% with Imidacloprid 21%	Bed bugs	2 mL/L	Bayer
Alfatek® 200 SC ^1^	Alpha-Cypermethrin 20%	Triatomines	5mL/L	Rogama
Ficam® VC ^2^	Bendiocarb 80%	mosquitoes, scorpions, sandflies, triatomines	7.5g/L	Bayer
Optigard® LT ^5^	Thiathomexam 25%	Flies (adults), Cockroaches	16g/L	Syngenta
Termidor® 25CE^6^	Fipronil 2.5%	Soil termites	15 mL/L	Basf

**Note: 1.** Pyrethroids; **2.** Carbamate; **3.** Pyrethroids associated with benzoylurea; **4.** Pyrethroids associated with neonicotinoids; **5.** Neonicotinoid; **6.** Phenylpyrazole.

The lack of a susceptibility reference lineage (SRL) for *C. lectularius* made it impossible to perform quantitative laboratory bioassays. To minimize this limitation, we used the discriminant dose for deltamethrin 30 times greater than that used by Romero et al.^6^. In response to this dose, the mortality results (6.6% to 30.0%) undoubtedly indicated the resistance of the studied populations to deltamethrin. However, considering the inexistence of SRL in the study with the formulated insecticides, in view of the observed mortality (0.0% to 83.0%), we referred to the populations as “indicative of resistance.” 

In this sense, ATB and PM bedbugs showed similarity in the resistance/susceptibility profile to the different insecticides tested (Kruskal-Wallis test, p = 0.94). The populations resistant to deltamethrin technical grade were indicative of resistance to all commercial insecticides tested, except for propoxur 1%. However, the different insecticides tested differed in effectiveness (Kruskal-Wallis test, p <0.05). No mortality was observed in the control groups (C1 and C2) (Table 2).

**TABLE 2:** Insecticide mortality (%) of *Cimex lectularius* collected in the homeless shelter “Unidade de Acolhimento Institucional para População de Rua - Albergue Tia Branca” and the migrant shelter “Unidade de Acolhimento Institucional para Migrantes - Pousadinha Mineira” Belo Horizonte, MG (Brazil).

Insecticides evaluated	*Unidade de Acolhimento Institucional*		*Unidade de Acolhimento Institucional*	
	*para População de Rua - Albergue Tia*		*para Migrantes - Pousadinha Mineira*	
	2014	2014	2015	2015
	N/Dead (%)	N/Dead (%)	N/Dead (%)	N/Dead (%)
Control Group 1 (C1)	30/0 (0.0)	30/0 (0.0)	30/0 (0.0)	30/0 (0.0)
Deltamethrin - technical grade	30/9 (30.0)	30/6 (20.0)	30/0 (0.0)	30/0 (0.0)
Control Group (C2) ^a^ to commercial insecticide formulations	30/0 (0.0)	30/0 (0.0)	30/0 (0.0)	30/0 (0.0)
Bifenthrin 20% ^G^	30/24 (80.0)	30/25 (83.3)	30/24 (80.0)	30/24 (80.0)
Pyrethrin 0.5% ^Ab^	30/1 (3.3)	30/0 (0.0)	30/1 (3.3)	30/0 (0.0)
Propoxur 1% ^H^	30/30 (100.0)	30/30 (100.0)	30/30 (100.0)	30/30 (100.0)
Alpha-Cypermethrin 3% With Flufenoxuron 3% ^Ab^	30/1 (3.3)	30/0 (0.0)	30/1 (3.3)	30/1 (3.3)
Beta-Cyfluthrin 10.5% With Imidacloprid 21% ^Bc^	30/0 (0.0)	30/2 (6.6)	30/1 (3.3)	30/1 (3.3)
Alpha-Cypermethrin 20% ^Bc^	30/0 (0.0)	30/0 (0.0)	30/2 (6.6)	30/2 (6.6)
Bendiocarb 80% ^F^	30/11 (36.6)	30/12 (40.0)	30/14 (46.6)	30/14 (46.6)
Thiathomexam 25% ^De^	30/3 (10.0)	30/5 (16.6)	30/5 (16.6)	30/5 (16.6)
Fipronil 2.5% ^Cd^	30/2 (6.6)	30/2 (6.6)	30/5 (16.6)	30/2 (6.6)

**Note: N.** Amostral number; **C1** - the papers impregnated with chloroform P.A. with Vaseline; **C2** - the papers treated with water; Statistical analysis of the effectiveness of commercial insecticides evaluated (Kruskal-Wallis p<0.05).

In the evaluation of commercial insecticide formulations recommended for the control of bed bugs in Brazil, *C. lectularius* was indicative of resistance to all insecticides (0 ≤ mortality ranging ≤ 83%); however, the mortality to bifenthrin was significantly higher than the rate found for other active ingredients. Although sodium-channel proteins are the site of action of all pyrethroids, it is believed that the difference found between bifenthrin and the other pyrethroids tested is due to the chemical structure and steric configuration of these insecticide molecules. The toxicity varies in response to the cis/trans molecule configuration, which is associated with the characteristics of the vehicle being used. Cis isomers, such as bifenthrin, show higher toxicity than the trans isomers present in other insecticide molecules^7^.

Although the pyrethroid resistance of *Cimex* has been common worldwide, there are no reports in Brazil. The pyrethroid resistance to *C. lectularius* has been attributed to cuticular thickening mechanisms, increased detoxifying enzyme activity, and mutations in sodium-channel proteins. As expected, the cross resistance of *C. lectularius* to pyrethroids and organochlorides has been reported. This can be easily understood because they have the same site of action, and organochlorides have been used for more than a decade without interruption until the replacement of this chemical group for carbamates^8^. 

Moreover, the continued use of insecticides belonging to the same class favors the selection of resistant specimens. This partly explains the reduced mortality rates found in laboratory bioassays with pyrethroids observed in this study. In public shelters where *C. lectularius* populations were collected, there was a massive use of the pyrethroid K-Othrine before and during the collections. Moreover, it should be noted that, in addition to the repetitive use of the same insecticide molecule, sub-doses and the lack of technical criteria for application of these pesticides result in operational failures that may have contributed to the selection of pyrethroid resistant insects^6^ ^-^ ^8^.

For neonicotinoids, the bedbug populations were indicative of resistance to thiathomexam 25% (10.0 ≤ mortality ≤ 16.6%). Neonicotinoids act selectively on the central nervous system of insects as nicotinic acetylcholine receptors (nAChRs), opening ion channels, causing depolarization and continuous electrical firing in post-synaptic neurons, resulting in paralysis and death. With the increased use of neonicotinoids, resistance to these insecticides has been increasingly observed^8^. Romero et al.^9^ found high levels of resistance in *C. lectularius* collected from households in Cincinnati and Michigan (USA) to four neonicotinoids recommended for bed bug control: acetamiprid, imidacloprid, dinotefuran, and thiamethoxam. There was an increase in alpha-esterase, beta-esterase, glutathione-S-transferase, and cytochrome P450. The authors believe that such resistance may be due to the pressure caused by the continuous use of neonicotinoid-based products in recent years. In addition to the increase in detoxifying enzymes, neonicotinoid resistance has been attributed to cuticular thickening^8^ and mutations in the subunits of nicotinic acetylcholine receptors^10^ ^,^ ^11^. The R81T mutation (arginine to threonine) present in the β1 nAChR subunit results in high levels of resistance to imidacloprid in the melon and cotton aphid, *Aphis gossypii* ^12^, and the green peach aphid, *Myzus persicae* ^13^. It should be noted that changes in nAChR have not yet been identified in cimicids. In Brazil, there are no reports of Cimicidae resistance to neonicotinoids.

The ATB and PM bedbugs were indicative of resistance to fipronil 2.5%, an insecticide belonging to the phenyl pyrazole group (6.6% ≤ mortality ≤ 16.6%). Phenyl pyrazoles are competitive antagonists of the neurotransmitter gamma-aminobutyric acid (GABA) that bind to its receptors, similar to benzene hexachloride and cyclodienes. Recently, resistance to the dieldrin gene was identified by decoding the genome of *C. lectularius* ^14^. It is a common target for phenyl pyrazoles (for example, fipronil). These data may provide important information on the resistance mechanism of the GABA receptor that is insensitive to cyclodienes and phenyl pyrazoles. 

Finally, the bedbugs studied were indicative of resistance to bendiocarb 80% (36.6% ≤ mortality ≤ 40%). In contrast, cimicids were susceptible to propoxur 1% (100% mortality). Although bendiocarb and propoxur are carbamates^8^, and both act on acetylcholinesterase, propoxur may have been effective because of the aerosol formulation of the product being used, linked to the characteristics of the insecticide molecule itself. In this sense, there may have been greater bioavailability of the active ingredient or synergism with other ingredients present in the aerosol formulation of propoxur 1%. Romero et al.^15^ found that the formulation in aerosol or direct spray on dry residues killed *C. lectularius* more quickly than the water-based formulation (Phanton SC). 

Several studies have reported resistance to carbamates and organophosphates in *C. lectularius*. The resistance to organophosphates and carbamates may be a result of insensitivity at the target site (acetylcholinesterase). Considering the molecular and enzymatic properties of CAChE1 and CAChE2, it has been suggested, based on *in vitro* assays, that CAChE1 is the most relevant toxicological target for organophosphates and carbamates in *C. hemipterus* ^8^. 

Considering the results obtained, field tests are recommended for the formulated insecticides. An evaluation of the fundamental aspects of the inclusion of these products in the control of bed bugs in the field, such as the characteristics of the area to be treated (size, physical features), properties of the insecticide molecules in the environment (e.g., toxicity to mammals and to the environment, residual products on different surfaces), logistical and operational aspects (e.g., frequency of application, surface treatment dynamics), as well as cost-effectiveness, is required. It is essential that for evaluation of these two products, chemical control activities should be performed simultaneously with environmental management. In addition, the identification of the mechanisms responsible for the insecticide-resistant phenotype observed in the study populations may be a useful tool for targeting alternative insecticides for controlling these insects.

Considering the similarity in the insecticide susceptibility profile of *C. lectularius* from ATB and PM linked to the large flow of users between these two shelters, it is possible that this is a single population maintained by passive dispersion in these environments. The analysis of the genetic structure of these insects is in progress, and will allow a better understanding of the factors responsible for the dispersion and/or maintenance of *C*. *lectularius* in these two shelters in Belo Horizonte.
